# Global Research Trends in Respiratory Syncytial Virus Vaccine Research: A Bibliometric Analysis

**DOI:** 10.3390/vaccines14100864

**Published:** 2026-09-30

**Authors:** Ana Raquel Batista de Carvalho, Glória Stéphany Silva de Araújo, Maria Eduarda Pereira Correia, Fabiana de Moura Souza, Telma Maria Evangelista de Araújo, Ana Raisla de Araújo Rodrigues, Andréia Rodrigues Moura da Costa Valle, Joaquim Guerra de Oliveira Neto, Rosilane de Lima Brito Magalhães, Daniela Reis Joaquim de Freitas, Maria Zélia de Araújo Madeira, Odinéa Maria Amorim Batista, José Couras da Silva Filho, Maria Eliete Batista Moura

**Affiliations:** 1 Uninovafapi University Center—Afya, Teresina 64049-550, Brazil; ana.raquel@afya.com.br; 2 Postgraduate Program in Nursing, Federal University of Piauí, Teresina 64049-550, Brazil; gloria.araujo@ufpi.edu.br (G.S.S.d.A.); eduardacorreia@ufpi.edu.br (M.E.P.C.); fabiana.souza@ufpi.edu.br (F.d.M.S.); telmaevangelista@ufpi.edu.br (T.M.E.d.A.); anaraisla@ufpi.edu.br (A.R.d.A.R.); andreiarmcvalle@hotmail.com (A.R.M.d.C.V.); joaquim.oliveira@ufpi.edu.br (J.G.d.O.N.); rosilane@ufpi.edu.br (R.d.L.B.M.); danielajrfreitas@ufpi.edu.br (D.R.J.d.F.); zeliamadeira15@yahoo.com.br (M.Z.d.A.M.); oenf@ufpi.edu.br (O.M.A.B.); 3 University Hospital, Federal University of Piauí, Teresina 64049-550, Brazil; jose.couras@hubrasil.gov.br

**Keywords:** respiratory syncytial virus, RSV vaccine, bibliometric analysis, science mapping, immunization, monoclonal antibodies

## Abstract

**Background/Objectives**: Respiratory syncytial virus (RSV) is a major cause of acute respiratory infections and has long posed challenges to vaccine development due to historical safety concerns and the complexity of protective immunity. Recent advances in structural biology, maternal immunization, vaccines for older adults, and monoclonal antibodies have reshaped the field. This study aimed to analyze global research trends in RSV vaccine research using a bibliometric approach. **Methods**: A bibliometric study was conducted using records retrieved from the Web of Science Core Collection and Scopus on 1 September 2026. After database merging, deduplication, and data refinement, bibliometric indicators, citation patterns, collaboration networks, source distribution, and intellectual structure were analyzed using Bibliometrix and Biblioshiny in R. **Results**: The final corpus comprised 2516 documents published between 1968 and 2026 across 646 sources, with an annual growth rate of 10.7%. A total of 9298 authors were identified, with an average of 7.08 coauthors per document, and 24.56% of publications involved international collaboration. Scientific production increased markedly from the 2010s onward. The United States remained the leading contributor, while the most recent period showed broader participation from other countries. Vaccine was the most productive source. Citation analyses highlighted historical publications on the formalin-inactivated RSV vaccine and subsequent advances related to the prefusion F protein as important components of the field’s intellectual structure. **Conclusions**: RSV vaccine research has evolved from a historically safety-constrained field into a mature translational area focused on prevention, implementation, real-world effectiveness, and equitable access.

## 1. Introduction

Respiratory syncytial virus (RSV) remains one of the leading etiological agents of acute respiratory infections worldwide, accounting for a substantial burden of morbidity and mortality, particularly among infants, older adults, and immunocompromised individuals. Although historically associated with the pediatric population, recent studies have shown that the impact of RSV among older adults is substantial, contributing to hospitalizations, exacerbations of chronic diseases, and increased mortality, particularly among individuals with cardiovascular, pulmonary, or immunological comorbidities. Global estimates indicate that the virus continues to represent an important challenge for health systems across different regions of the world [1,2].

The development of preventive strategies against RSV was marked by scientific obstacles that delayed the achievement of effective immunogens for several decades. The experience with the formalin-inactivated RSV vaccine (FI-RSV) in the 1960s resulted in cases of enhanced respiratory disease following natural exposure to the virus, becoming one of the most relevant episodes in the history of vaccinology. In parallel, biological characteristics of RSV, including the frequent occurrence of reinfections and the complexity of the immune response induced by natural infection, hindered the development of strategies capable of providing durable and safe protection [3,4].

This scenario began to change with advances in the structural understanding of the RSV F glycoprotein. The identification of the prefusion conformation of the protein enabled the recognition of highly neutralizing epitopes and provided the basis for the rational development of new vaccine platforms. Based on these discoveries, different approaches began to be investigated, including recombinant vaccines, messenger RNA-based vaccines, maternal immunization, and long-acting monoclonal antibodies [5,6].

The results of these advances have become evident in recent years. Phase III clinical trials have demonstrated high efficacy of vaccines based on the prefusion F protein in preventing RSV-associated lower respiratory tract disease in older adults and in infants protected through maternal immunization. Simultaneously, monoclonal antibodies such as nirsevimab have shown consistent results in reducing RSV-related hospitalizations in young children. The incorporation of these strategies into clinical practice has significantly expanded the number of studies focused on effectiveness under real-world conditions, epidemiological surveillance, safety, economic evaluation, and the implementation of immunization programs [7,8,9,10,11,12,13,14].

As a scientific field expands, it becomes necessary to understand not only the results produced but also how knowledge is constructed, disseminated, and organized. In this regard, bibliometric studies are important tools for assessing publication patterns, identifying influential authors and institutions, mapping scientific collaboration networks, and recognizing emerging research trends. Unlike narrative and systematic reviews, which focus on synthesizing clinical or experimental evidence, bibliometrics allows the structure and dynamics of scientific production to be examined in a quantitative and reproducible manner [15,16,17].

Despite the rapid expansion of the literature on RSV vaccines, analyses capable of integrating indicators of scientific productivity, the intellectual structure of the field, international collaboration networks, and the thematic evolution of research from a global perspective remain scarce. This gap becomes particularly relevant following the approval of the first vaccines and monoclonal antibodies for preventing infection, a period marked by profound changes in the scientific agenda related to RSV. Understanding these patterns may provide a structured overview of the development of the field, identify areas receiving greater or lesser scientific attention, and reveal gaps in the geographical, institutional, and collaborative distribution of the available literature.

In this context, the present study aimed to analyze global trends in scientific production on vaccines against respiratory syncytial virus and to investigate patterns of productivity, scientific collaboration, and intellectual structure in this research field.

## 2. Materials and Methods

### 2.1. Study Design

This is a bibliometric study with a descriptive design, quantitative approach, and bibliographic nature. Bibliometrics enables the quantitative examination of scientific literature, allowing the identification of publication patterns, academic productivity, scientific impact, collaboration networks, and thematic trends in a given field of knowledge [18].

In addition to assessing scientific performance, bibliometric studies support the exploration of the intellectual, conceptual, and social structure of the literature through science mapping techniques, such as co-citation, co-authorship, bibliographic coupling, and keyword co-occurrence analyses. These approaches contribute to identifying influential authors, emerging topics, collaborative relationships, and the thematic evolution of research over time [19].

The methodological conduct of this study was based on the recommendations proposed by Donthu et al. [20], who describe five essential stages for bibliometric analyses: defining the research question, selecting the database, developing the search strategy, extracting and analyzing the data, and, finally, interpreting and presenting the results. In parallel, the principles of science mapping described by Zupic and Čater [18] were considered, especially with regard to the relational analyses used to map the scientific and intellectual structure of research fields.

The development and reporting of this study followed the recommendations of the Preferred Reporting Items for Bibliometric Analysis (PRIBA) [21], which are aimed at ensuring transparency, standardization, and reproducibility in bibliometric studies in the health field. Additionally, the BIBLIO guideline for biomedical bibliometric reviews was considered, particularly regarding the detailed description of the search strategy, data refinement, analytical procedures, and structured presentation of results [22].

The guiding question of this study was: What are the bibliometric indicators related to vaccines against respiratory syncytial virus?

### 2.2. Data Source and Search Strategy

The bibliographic search was conducted in the Web of Science™ Core Collection (WoSCC) (Clarivate, Londres, Reino Unido) and Scopus^®^ databases (Elsevier, Amsterdã, Países Baixos) on 1 September 2026, covering all documents indexed up to the date of data collection. The inclusion of two databases with broad international coverage aimed to increase the representativeness of the retrieved scientific output and reduce limitations associated with the use of a single data source, as Web of Science and Scopus differ in their coverage of journals, subject areas, and the geographic distribution of scientific output [23].

To reduce potential biases related to continuous database updates and temporal fluctuations in citation counts, all records were extracted on a single day, a strategy recommended to increase the stability and reproducibility of bibliometric analyses.

The search strategy was developed by combining controlled and uncontrolled descriptors related to the topic. For this purpose, terms indexed in Medical Subject Headings (MeSH) were used, complemented by free-text keywords and terminological variations frequently employed in the scientific literature. The terms were combined using the Boolean operators AND and OR, with the application of truncation symbols (*) when appropriate.

The search was conducted in the Title (TI) field to ensure greater specificity and thematic alignment of the retrieved sample. Title-based search strategies have been employed in bibliometric studies to increase the thematic precision of the retrieved set and reduce the inclusion of documents in which the terms of interest are mentioned only incidentally in the abstract or keywords.

This approach, however, entails a trade-off between specificity and sensitivity, as relevant studies that do not explicitly include the searched terms in the title may not be retrieved. Thus, the use of the title field was adopted as a strategy oriented toward the specificity of the bibliometric corpus, while preserving greater thematic coherence among the analyzed documents [24,25].

The search strategy used was: (“respiratory syncytial virus” OR RSV OR HRSV) AND (vaccine* OR vaccination OR immunization OR immunisation), adapted to the syntax of each database, using the TITLE field in Scopus and TI in Web of Science. No filters by document type were applied in order to preserve the diversity of scientific communication formats retrieved by the databases and provide a more comprehensive representation of the literature related to the topic [26].

The search initially retrieved 2224 records from the Web of Science Core Collection and 2072 records from Scopus, totaling 4296 records before database integration. After merging the datasets, 1770 duplicate records were identified and removed. An additional verification based on DOI identified nine residual duplicates, which were also removed.

**Figure 1:**
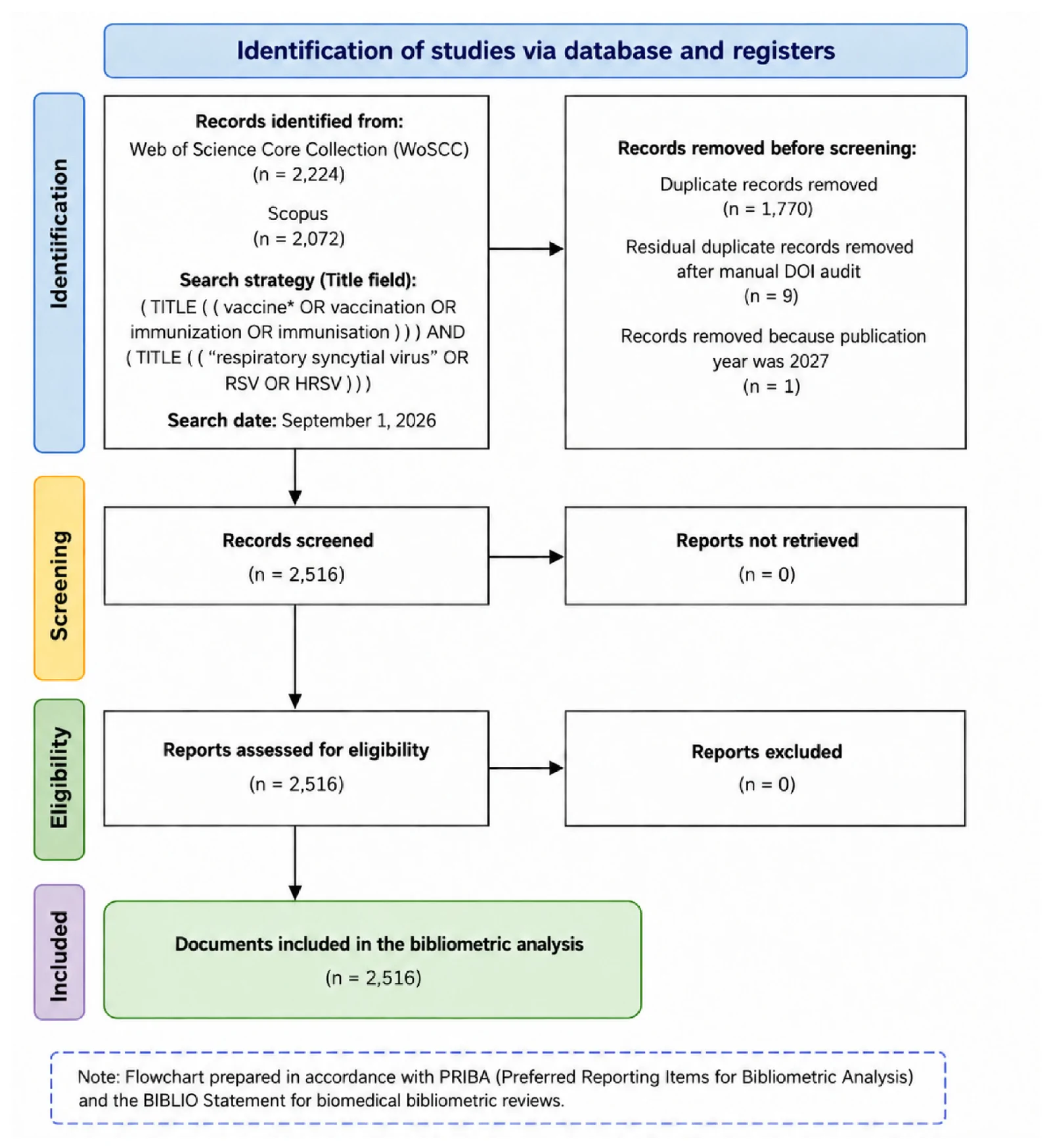
PRISMA flow diagram for article selection.

In addition, one document assigned to the publication year 2027 was excluded because it fell outside the period defined for the analysis. At the end of the integration, deduplication, and refinement processes, 2516 documents comprised the corpus of the bibliometric analysis. The process of study identification, selection, and inclusion was presented in a flowchart, as recommended by the PRIBA and BIBLIO guidelines (Figure 1).

### 2.3. Data Analysis

The records were exported from the Web of Science Core Collection in BibTeX (.bib) format, with Full Record and Cited References, and from Scopus in CSV format, including all available metadata and cited references. This configuration enabled the comprehensive retrieval of the metadata required for the bibliometric analyses, including authors, titles, abstracts, keywords, journals, institutions, countries, number of citations, and cited references. The selection of this export configuration is widely recommended in scientific mapping studies, as it ensures greater data integrity for network analyses and bibliometric indicators [18,19].

After the extraction stage, the records underwent a process of data refinement and standardization, including the identification of duplicates, exclusion of inconsistent records, and normalization of institutional names when necessary. This step is considered essential in bibliometric reviews, particularly for term co-occurrence analyses and the construction of thematic networks, because spelling variations, synonyms, abbreviations, and terminological inconsistencies may directly affect the structure and interpretation of science maps [20].

The bibliometric analyses were conducted in Rstudio version 2023.06.2 (Posit Software, PBC, Boston, MA, USA), using the R statistical environment, version 4.4.3 (R Foundation for Statistical Computing, Vienna, Austria), and the Bibliometrix version 4.3.3 (University of Naples Federico II, Naples, Italy) package through its graphical interface, Biblioshiny. Bibliometrix was used because it provides an integrated workflow for importing, organizing, analyzing, and visualizing bibliographic data, allowing descriptive analyses, scientific productivity indicators, collaboration networks, and conceptual mapping to be performed in a reproducible and systematic manner [19].

In addition to descriptive analyses of scientific productivity, analyses of the intellectual, social, and conceptual structure of the investigated field were performed, including collaboration networks among countries, authors, and institutions; co-citation analyses of authors and documents; and bibliographic coupling. These approaches make it possible to understand not only the quantitative evolution of scientific production but also the formation of research communities, the main thematic cores, and emerging trends over time [20,21].

## 3. Results

**Figure 2:**
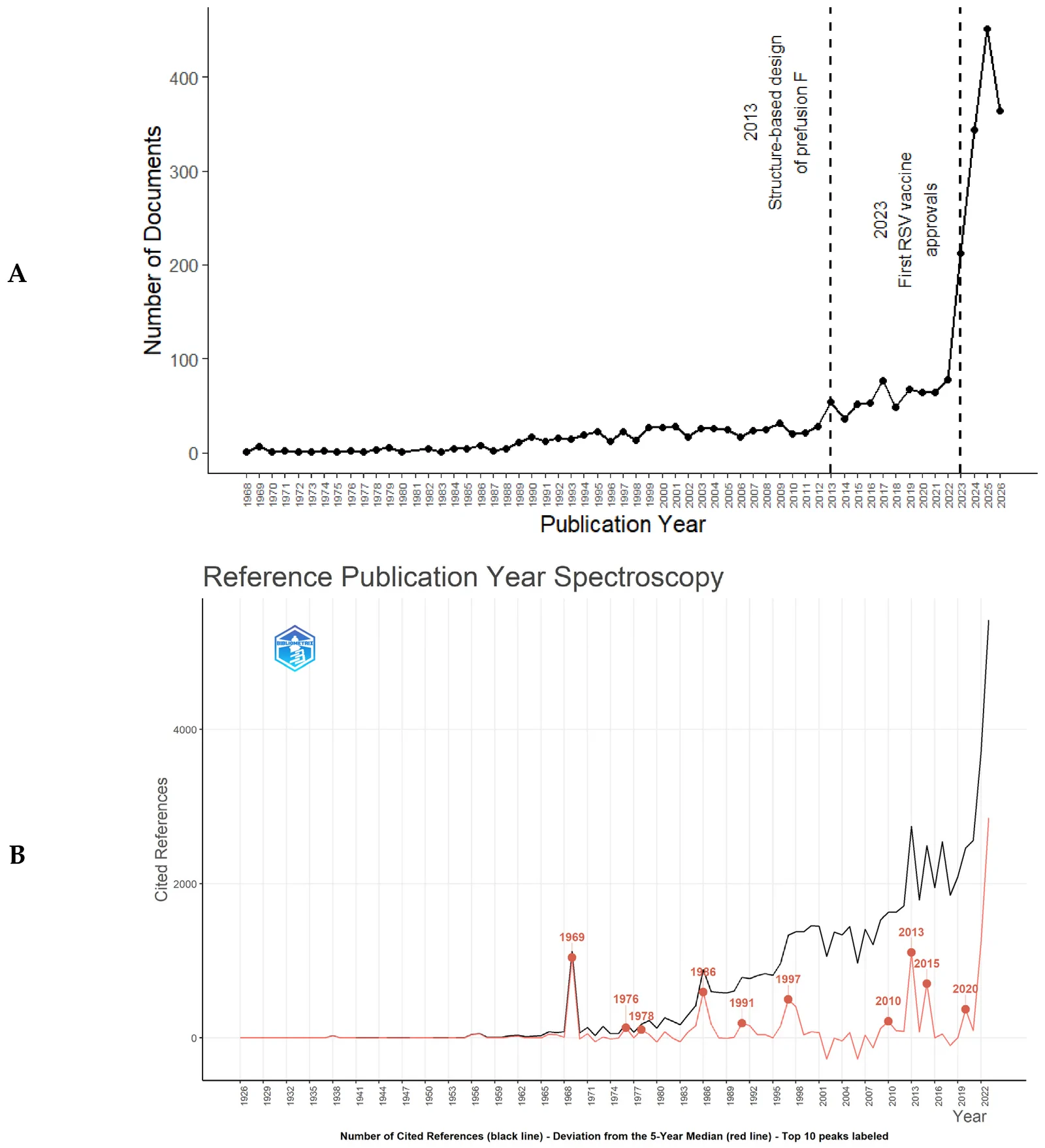

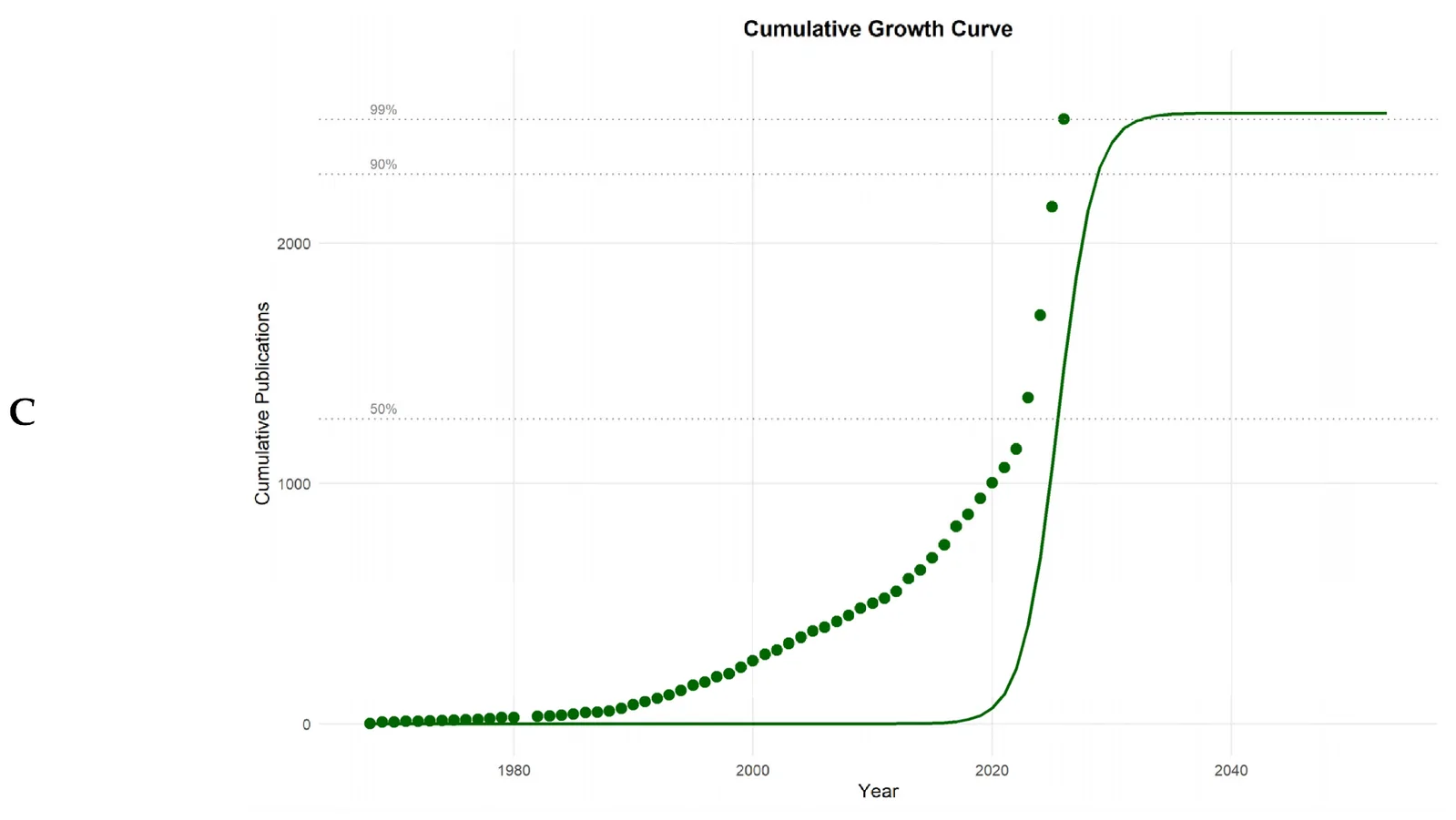
Temporal dynamics of scientific production on vaccines against respiratory syncytial virus. (**A**) Annual scientific production, with selected milestones in RSV vaccine development. (**B**) Reference publication year spectroscopy (RPYS), showing the distribution of cited references by publication year and deviations from the five-year median. (**C**) Cumulative growth of scientific publications, with observed data and the fitted growth curve.

As presented in Figure 2, the analysis included 2516 documents published between 1968 and 2026, distributed across 646 sources. Scientific production showed an annual growth rate of 10.7%, with a mean document age of 8.39 years and a mean of 20.42 citations per document. A total of 9298 authors and 17,803 authorship occurrences were identified. The mean number of co-authors per document was 7.08, and 24.56% of the publications involved international collaboration.

The corpus comprised different types of documents, with articles predominating (*n* = 1459), followed by conference proceedings (*n* = 292), reviews (*n* = 216), editorial materials (*n* = 169), and notes (*n* = 104).

Low scientific production was observed during the first decades of the analyzed period, followed by gradual growth beginning in the 2000s and more pronounced acceleration after 2010. In Figure 2A, the milestones of 2013, corresponding to the consolidation of the structural design based on the F protein in its prefusion conformation, and 2023, the year of the first approvals of RSV vaccines, were highlighted to provide temporal context for the expansion of the literature.

Reference Publication Year Spectroscopy (RPYS) analysis identified peaks in 1969, 1976, 1978, 1986, 1991, 1997, 2010, 2013, 2015, and 2020, indicating years in which published references exhibited citation frequencies above the local trend of the series, reflecting greater influence on the intellectual structure of the field without necessarily implying greater clinical impact (Figure 2B).

The cumulative growth curve demonstrated a progressive expansion of the literature, particularly from the 2010s onward, confirming the recent concentration of scientific production on RSV vaccines (Figure 2C).

**Figure 3:**
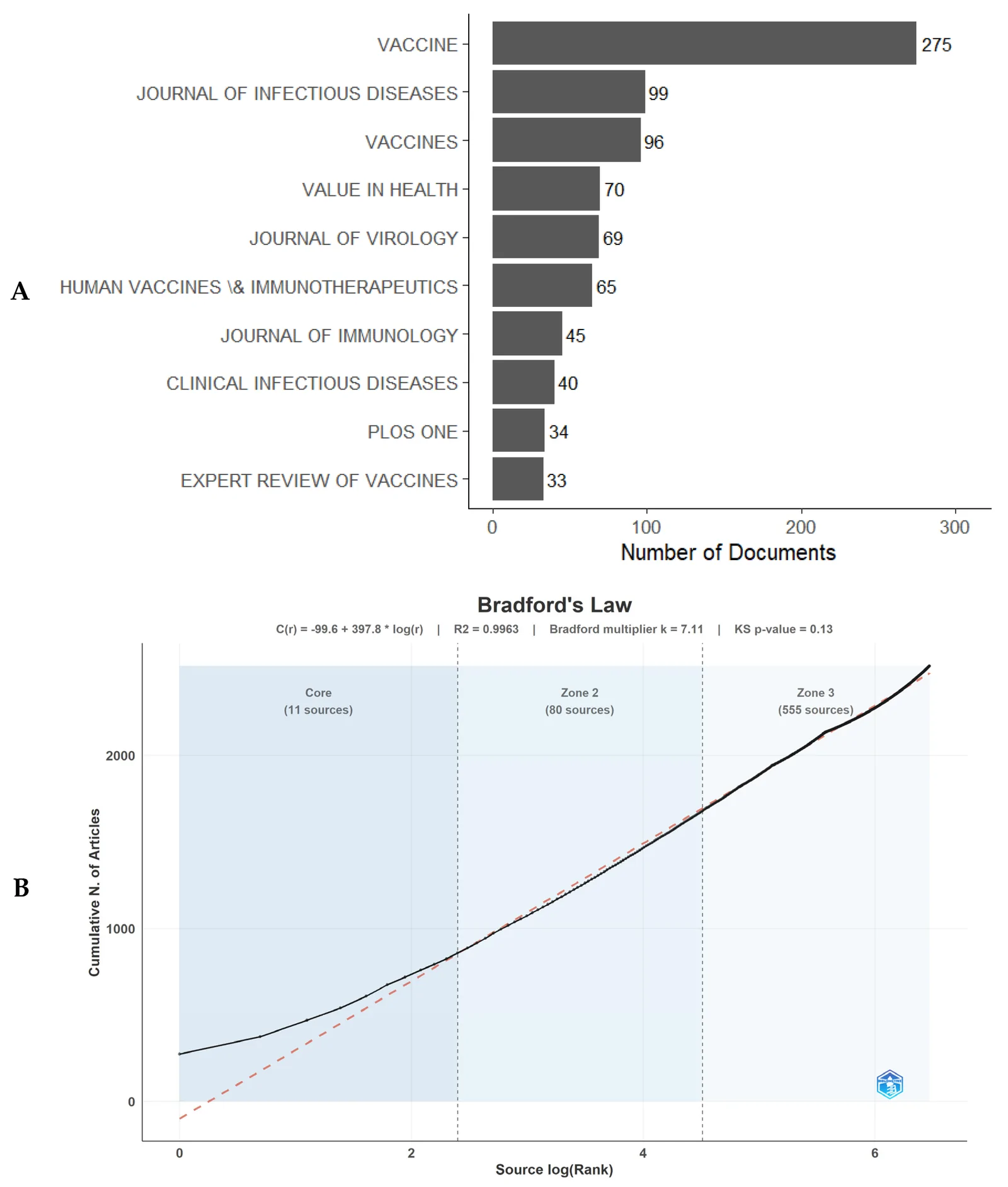
Productivity and distribution of scientific sources on vaccines against respiratory syncytial virus. (**A**) Most productive publication sources according to the number of documents; (**B**) distribution of publication sources according to Bradford’s Law. In Figure 3B, the solid black line shows the observed cumulative number of articles, and the dashed red line represents the logarithmic fit. The vertical dashed gray lines mark the boundaries between Bradford zones. Blue shading, from darkest to lightest, identifies the core (11 sources), Zone 2 (80 sources), and Zone 3 (555 sources).

Scientific production on RSV vaccines was distributed across 646 publication sources. Vaccine was the most productive source, with 275 documents, followed by the Journal of Infectious Diseases (*n* = 99) and Vaccines (*n* = 96) (Figure 3A). Bradford’s Law analysis identified a core comprising 11 sources, corresponding to 1.7% of the total, which accounted for 858 documents (34.1%). The second zone comprised 80 sources and 821 documents (32.6%), whereas the third zone included 555 sources and 837 documents (33.3%).

**Figure 4:**
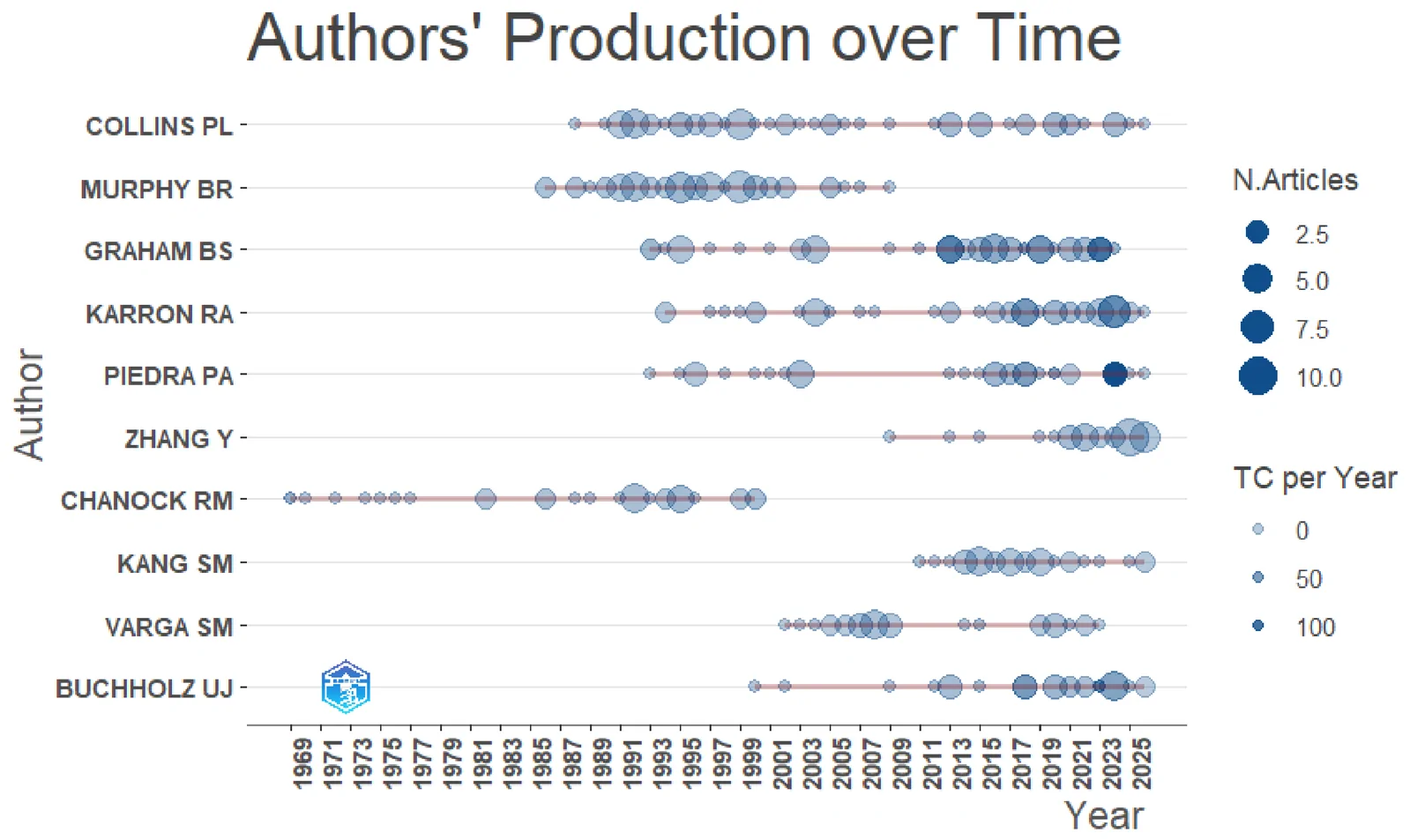
Authors’ publication patterns over time. Red line: The author collaboration network revealed distinct co-authorship clusters (Figure 5), indicating that RSV vaccine research is organized into multiple collaborative groups rather than a single homogeneous community.

Scientific production on vaccines against respiratory syncytial virus was conducted by 9298 unique authors. The most productive researchers were Sterud T, with 47 publications, followed by Graham BS, Karron RA, and Murphy BR, with 38 documents each. The temporal analysis showed that some of the most productive authors concentrated their contributions in recent years, especially between 2020 and 2024 (Figure 4).

**Figure 5:**
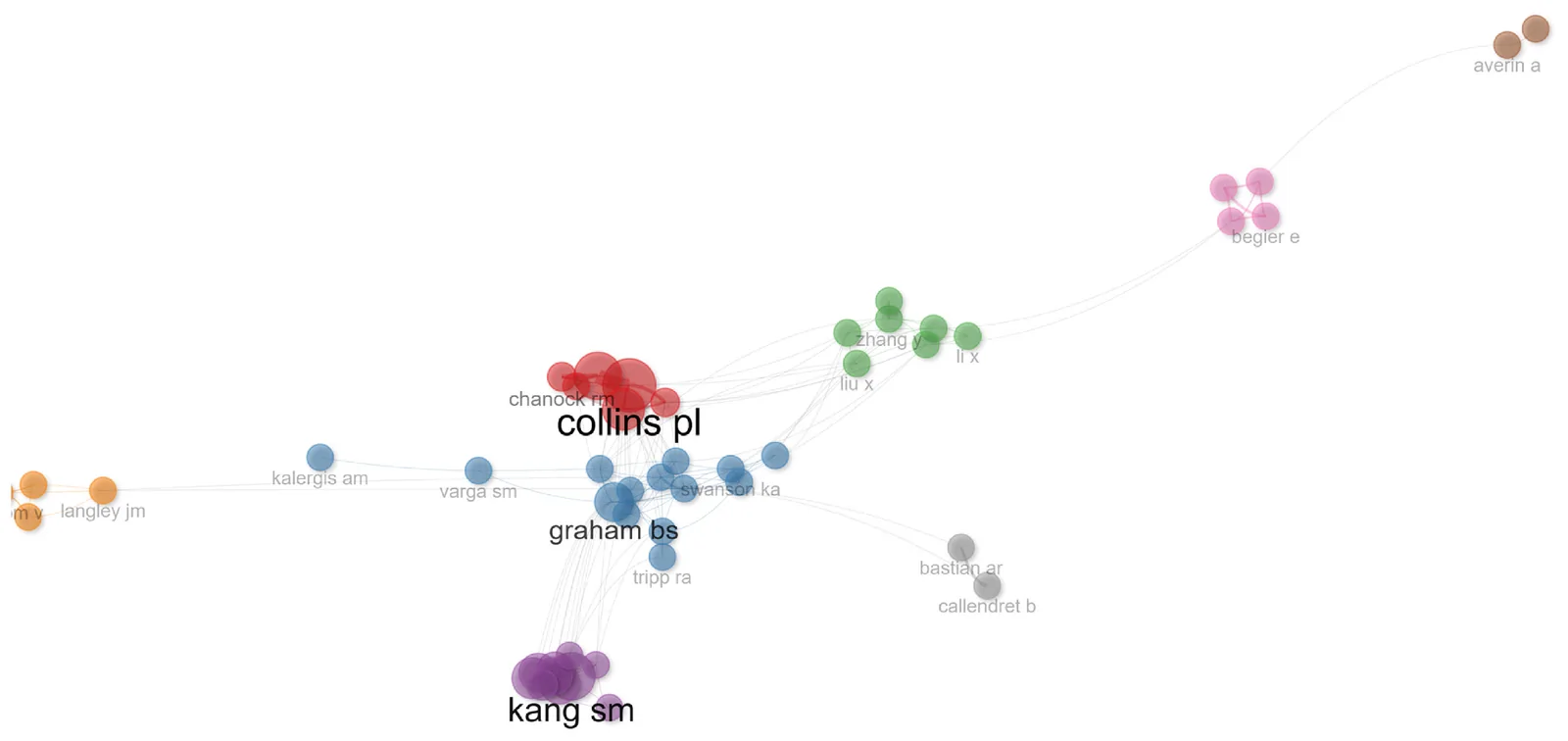
Collaboration network among authors and scientific communities.

The author collaboration network revealed several distinct co-authorship clusters (Figure 5), indicating that RSV vaccine research is organized into multiple collaborative groups rather than a single homogeneous community.

The analysis of scientific production by country showed the leadership of the United States, with 817 documents, corresponding to 32.5% of the total production (Table 1). This was followed by China (167 documents; 6.6%), the United Kingdom (121; 4.8%), Canada (104; 4.1%), and the Netherlands (91; 3.6%).

The United States remained the leading contributor across all periods, with its share increasing from 47.3% in 1968–1999 to 55.7% in 2013–2022, followed by a decrease to 38.9% in 2023–2026. In the most recent period, China and Germany accounted for 10.6% and 10.5%, respectively, while Belgium, Italy, and Australia also showed greater participation than in earlier periods (Table 2).

The affiliations with the highest number of publications on RSV vaccine research were Vanderbilt University (*n* = 100) and the University of Saskatchewan (*n* = 91), followed by Baylor College of Medicine (*n* = 82), the University of Oxford (*n* = 82), Emory University (*n* = 80), and the University of Iowa (*n* = 80) (Table 3).

**Figure 6:**
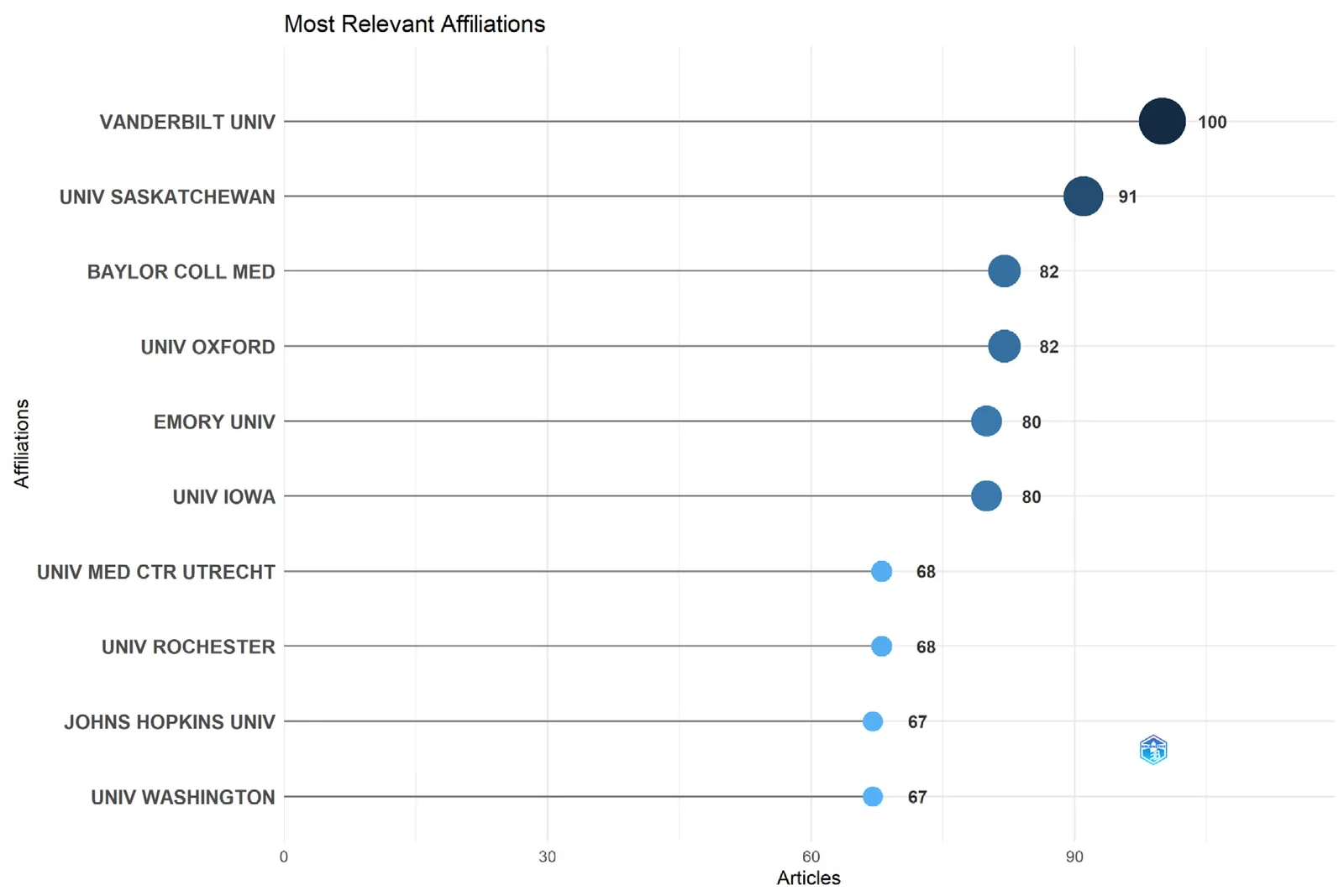
Most productive affiliations in the literature on vaccines against respiratory syncytial virus. The shades of blue indicate the number of articles associated with each institution: darker shades represent higher publication counts. Larger circles also indicate higher counts.

Vanderbilt University showed its highest relative contribution in 1968–1999 (5.9%), while Emory University reached its highest share in 2013–2022 (5.2%). In the most recent period, Harvard Medical School showed the highest relative participation among the listed institutions (2.6%), followed by the University of Rochester and the University of Oxford (1.6% each) (Figure 6).

The coexistence of historical articles published in the 1960s and 1990s was observed. The most cited work was that of Kim et al. (1969) [27], published in the American Journal of Epidemiology, with 1700 total citations, followed by McLellan et al. (2013) [28], published in Science, with 915 citations (Table 4).

## 4. Discussion

Respiratory syncytial virus vaccine research has followed a trajectory strongly influenced by the historical challenges of vaccine development. The experience with the formalin-inactivated RSV vaccine in the 1960s, which was associated with enhanced respiratory disease following subsequent natural infection, established a long-lasting safety concern in the field [28,31,33].

This episode influenced subsequent approaches to vaccine development, particularly regarding the quality of the immune response, immunopathology, and the safety evaluation of new vaccine candidates [37,38,39]. Rather than representing only a historical event, the FI-RSV experience remained part of the intellectual structure of the field, as also indicated by the persistence of publications from 1969 among the major peaks identified in the RPYS analysis.

The temporal pattern identified in this study shows that scientific production expanded gradually over several decades and accelerated more markedly from the 2010s onward. This growth occurred during a period of major advances in RSV research, particularly the structural characterization of the F protein in its prefusion conformation and the subsequent development of new preventive platforms [3,4,29]. However, the increase in publication output should not be attributed exclusively to developments specific to RSV.

A broader expansion of scientific production has also been documented across medical and health sciences, reflecting the long-term growth of the biomedical literature [40]. Thus, the recent acceleration observed in RSV vaccine research likely reflects the convergence of a general expansion in biomedical publishing with field-specific scientific and clinical developments.

The RPYS analysis reinforces the persistence of historical milestones in the intellectual structure of RSV vaccine research. The studies by Kim, Chin, and Fulginiti, published in 1969, remain prominent because they documented enhanced respiratory disease in children previously immunized with the formalin-inactivated RSV vaccine [28,31,33]. Their continued presence among the major reference-year peaks indicates that these publications remain influential in how subsequent RSV vaccine research has been framed, particularly with regard to vaccine safety, immunopathology, and the interpretation of immune responses [38,39]. However, this bibliometric prominence should be interpreted as evidence of sustained scientific visibility and intellectual influence rather than as a direct measure of current clinical relevance or methodological quality.

The persistence of these historical references suggests that vaccine safety remained an organizing concern throughout the development of the field. Subsequent research incorporated not only measures of immunogenicity, but also the functional quality of antibody responses, inflammatory mechanisms, and the potential for vaccine-associated enhanced disease [38,39]. In this sense, the FI-RSV experience continued to inform the scientific questions addressed by later vaccine platforms, linking early safety concerns to the immunological and clinical evaluation of contemporary candidates. At the same time, citation-based indicators should be interpreted in light of publication age, since older studies have had considerably more time to accumulate citations than recently published clinical trials [41].

The structural characterization of the RSV F protein in its prefusion conformation represented an important transition in vaccine development by providing a basis for structure-guided immunogen design. McLellan et al. demonstrated that stabilization of the prefusion F protein could preserve highly neutralization-sensitive epitopes and support the development of more immunogenic vaccine candidates [29]. In the present bibliometric analysis, the prominence of this publication among the most cited documents and its association with the 2013 RPYS peak indicate sustained scientific visibility and support its interpretation as an important intellectual milestone in the transition toward structure-based RSV vaccine development [3,29].

The subsequent publication of large clinical trials in pregnant women and older adults illustrates the broadening of the populations targeted by RSV preventive strategies [30,32,34,35]. These studies expanded the clinical agenda beyond vaccine development itself to include efficacy in specific risk groups, duration of protection, safety, and applicability across different clinical contexts. More recent research has also incorporated real-world effectiveness, pharmacovigilance, and passive immunoprophylaxis [42,43,44]. However, because the present bibliometric analysis did not classify the 2,516 documents according to the populations studied, these developments should not be interpreted as a quantitative demonstration of changes in study populations over time, but rather as evidence of diversification in the recent clinical literature.

Scientific production on RSV vaccines was distributed across 646 publication sources, with *Vaccine* as the most productive source, followed by the *Journal of Infectious Diseases* and *Vaccines*. This distribution indicates that RSV vaccine research is concentrated in a relatively small group of recurrent publication venues while also extending across a broad range of additional sources. The prominence of journals focused on vaccinology and infectious diseases is consistent with the clinical and translational orientation of the field, whereas the wider dispersion of publications reflects the multidisciplinary nature of RSV research.

Bradford’s Law further illustrates this pattern of concentration and dispersion. The core comprised 11 sources accounting for 34.1% of all documents, while the second and third zones included 80 and 555 sources, respectively, each contributing approximately one third of the total production. This distribution indicates the coexistence of a specialized editorial core with substantial dissemination across peripheral sources, consistent with the structure described by Bradford’s Law [45].

The co-authorship network provides a complementary view of the social structure of RSV vaccine research by showing how authors are connected through shared publications. Rather than forming a single homogeneous network, the field is organized into several collaborative groups, with more densely connected components and smaller peripheral clusters. This configuration indicates that scientific production has developed through multiple research communities linked by different degrees of collaboration. The structure of the network should, however, be interpreted as evidence of formal co-authorship relationships and not as a direct measure of scientific influence, innovation, research quality, or clinical impact [18,19].

International collaboration accounted for 24.56% of the publications included in this study and should be interpreted within the broader internationalization of biomedical research. Previous analyses have documented increasing cross-country participation and collaboration in biomedical publications, although direct numerical comparisons with the present findings are limited by differences in study periods, databases, document selection, and definitions of international collaboration [46,47]. The collaborative pattern observed in RSV vaccine research therefore appears to accompany broader movement toward increasingly international biomedical research rather than representing a phenomenon unique to this field.

The geographical distribution of RSV vaccine research remained concentrated in a relatively limited group of countries, although the temporal analysis indicates a broadening of participation over time. The United States remained the leading contributor throughout the study period, but its relative share decreased from 55.7% in 2013–2022 to 38.9% in 2023–2026, while countries such as China, Germany, Belgium, Italy, and Australia increased their participation. This pattern should be interpreted as a diversification of the geographical base of scientific production rather than as a decline in US research activity, since relative participation may decrease even when absolute output continues to grow. Nevertheless, the leading contributors remain concentrated in countries with substantial scientific and technological capacity. This distribution contrasts with the epidemiological burden of RSV, particularly in children, which remains disproportionately concentrated in low- and middle-income settings [47].

The geographical mismatch between the production of scientific evidence and the burden of RSV has implications beyond publication volume. Evidence generated predominantly in settings with greater health-system resources may not fully capture differences in seasonality, access to prenatal care, surveillance capacity, affordability, acceptability, and implementation of preventive strategies in other contexts. This issue has become increasingly relevant with the availability of maternal vaccination and long-acting monoclonal antibodies, for which implementation depends not only on efficacy but also on financing, supply, delivery infrastructure, and local epidemiology [48,49]. Expanding research participation in underrepresented settings is therefore important for generating evidence that is applicable to populations and health systems bearing a substantial proportion of the disease burden.

Changes in the institutional distribution of publications over time reveal that the organizational basis of RSV vaccine research has also shifted across the different stages of the field. Vanderbilt University had greater relative participation in the earliest period, whereas Emory University and the University of Georgia reached their highest shares between 2013 and 2022.

In the most recent period, Harvard Medical School increased its relative contribution, while the University of Oxford and the University of Rochester remained among the institutions with greater participation. Rather than indicating a fixed hierarchy of institutions, this temporal variation reflects changes in the groups and research centers contributing to the development of RSV vaccine research over time. Institutional frequencies, however, should be interpreted as patterns of participation in scientific production and not as direct measures of institutional leadership, research quality, or clinical impact.

The increasing incorporation of long-acting monoclonal antibodies into RSV prevention requires a clear distinction between active vaccination and passive immunoprophylaxis. Unlike vaccines, which induce an adaptive immune response, monoclonal antibodies provide immediate passive protection and therefore constitute a different preventive approach. Their inclusion in the recent RSV prevention landscape nevertheless reflects the expansion of available strategies beyond conventional vaccination, with different options being considered according to age, clinical risk, seasonality, access, and health-system capacity [50,51].

As vaccines and monoclonal antibodies become more widely used, questions that cannot be fully addressed under controlled trial conditions assume greater importance. Effectiveness in routine practice, duration of protection, rare adverse events, uptake, cost-effectiveness, and implementation across different health systems are particularly relevant because preventive options differ in their target populations, timing of administration, delivery requirements, and financial implications [51,52].

For infants, these questions include the comparative use of maternal vaccination and long-acting monoclonal antibodies under different epidemiological and programmatic conditions, whereas studies involving older adults need to account for multimorbidity, frailty, and heterogeneity in baseline risk. The generation of evidence within low- and middle-income settings is especially important, since findings produced predominantly in better-resourced health systems may not fully represent local epidemiology, access, or implementation constraints [8,53].

Given the rapid expansion of RSV prevention and implementation research following the introduction of new preventive interventions, the bibliometric structure described here should be considered time-dependent. Future updates may modify the collaboration patterns, institutional participation, and distribution of research topics observed in the present analysis as the post-licensure literature continues to expand.

Within this changing research landscape, bibliometric analysis can contribute by showing where scientific attention has been concentrated, how patterns of collaboration and institutional participation have evolved, and which countries or settings remain less represented in the literature. These indicators should not be interpreted as measures of the clinical superiority of specific preventive strategies, but they are useful for identifying gaps in the distribution of scientific evidence and for contextualizing priorities for subsequent clinical, epidemiological, and implementation research.

The findings of this study should be interpreted in light of limitations related both to bibliometric methods and to the methodological choices adopted in the analysis. The combined use of Web of Science Core Collection and Scopus was intended to increase coverage of the literature, although differences in indexing practices, citation records, and metadata structure between the databases cannot be completely eliminated. Restricting the search to the title field increased thematic specificity but may have reduced sensitivity by excluding relevant publications in which RSV vaccination was addressed only in the abstract or keywords.

The year 2026 was also incomplete at the time of data collection, which was conducted on September 1, and its publication count should therefore not be interpreted as evidence of a decline in scientific activity. Citation-based indicators are additionally influenced by publication age and database coverage and primarily represent scientific visibility rather than methodological quality, clinical relevance, or impact on health policy.

## 5. Conclusions

This bibliometric analysis showed a substantial expansion of RSV vaccine research, particularly from the 2010s onward, in parallel with major advances related to the prefusion F protein and the development of new preventive strategies. The intellectual structure of the field remains strongly influenced by the historical experience with the formalin-inactivated RSV vaccine, while more recent clinical literature reflects the expansion of prevention strategies to include maternal vaccination, immunization of older adults, and passive immunoprophylaxis. Scientific production is concentrated in a relatively small core of publication sources and remains led by a limited group of countries and institutions, although the most recent period shows broader geographical participation and an increasingly international pattern of collaboration.

The geographical imbalance in scientific production remains an important limitation of the evidence base, particularly because a substantial proportion of the RSV disease burden occurs in settings that are still underrepresented in the literature. As vaccines and monoclonal antibodies move into broader clinical use, future research should expand evidence on real-world effectiveness, post-licensure safety and surveillance, implementation across different health systems, health-economic evaluation, equitable access, and the population-level impact of preventive interventions. Continued bibliometric monitoring may help identify changes in collaboration, institutional participation, and the geographical distribution of research, while highlighting areas in which locally applicable evidence remains limited.

## Figures and Tables

**Table 1:** Scientific production and collaboration pattern of the leading countries.

Country	Articles	Articles %	SCP	MCP	MCP %
USA	817	32.47	671	146	17.87
CHINA	167	6.64	150	17	10.18
UNITED KINGDOM	121	4.81	68	53	43.80
CANADA	104	4.13	72	32	30.77
NETHERLANDS	91	3.62	54	37	40.66
FRANCE	66	2.62	46	20	30.30
GERMANY	62	2.46	39	23	37.10
BELGIUM	58	2.31	13	45	77.59
AUSTRALIA	51	2.03	35	16	31.37

Abbreviations: SCP, Single-Country Publications; MCP, Multiple-Country Publications.

**Table 2:** Temporal distribution of the leading countries in RSV vaccine research, according to share of documents (%).

Country	1968–1999	2000–2012	2013–2022	2023–2026
United States	47.3%	51.0%	55.7%	38.9%
United Kingdom	9.3%	6.7%	12.3%	10.5%
China	0.0%	3.8%	8.9%	10.6%
Canada	11.0%	9.2%	6.6%	6.5%
Germany	0.4%	2.9%	4.5%	10.5%
Netherlands	0.8%	8.3%	8.2%	5.4%
Belgium	1.3%	1.9%	5.1%	7.7%
France	3.4%	11.5%	3.5%	4.6%
Italy	0.4%	0.6%	3.2%	5.5%
Australia	0.0%	2.2%	4.2%	4.6%

**Table 3:** Temporal distribution of the leading institutions in RSV vaccine research, according to share of documents (%).

Institution	1968–1999	2000–2012	2013–2022	2023–2026
University of Rochester	4.2%	1.9%	2.4%	1.6%
Vanderbilt University	5.9%	3.8%	1.0%	1.3%
Baylor College of Medicine	3.0%	2.2%	3.5%	0.7%
Emory University	0.0%	1.9%	5.2%	0.5%
University of Oxford	0.0%	0.0%	2.9%	1.6%
Johns Hopkins University	3.0%	2.5%	0.5%	1.5%
Harvard Medical School	0.0%	0.0%	0.2%	2.6%
University of Colorado	0.8%	1.0%	3.0%	0.8%
University of Georgia	0.0%	2.5%	3.5%	0.3%
University Medical Center Utrecht	0.0%	0.6%		

**Table 4:** Ranking of the Top 10 most cited articles published on the subject.

Author, Year, Journal	Title	Total Citations	TC per Year
KIM HW, 1969, AM J EPIDEMIOL [27]	Respiratory Syncytial Virus Disease in Infants Despite Prior Administration of Antigenic Inactivated Vaccine	1700	29.3
MCLELLAN JS, 2013, SCIENCE [28]	Structure-Based Design of a Fusion Glycoprotein Vaccine for Respiratory Syncytial Virus	915	65.4
KAMPMANN B, 2023, NEW ENGL J MED [29]	Bivalent Prefusion F Vaccine in Pregnancy to Prevent RSV Illness in Infants	848	212
PAPI A, 2023, NEW ENGL J MED [30]	Respiratory Syncytial Virus Prefusion F Protein Vaccine in Older Adults	677	169.2
WALSH EE, 2023, NEW ENGL J MED [31]	Efficacy and Safety of a Bivalent RSV Prefusion F Vaccine in Older Adults	578	144.5
CHIN J, 1969, AM J EPIDEMIOL [32]	Field Evaluation of a Respiratory Syncytial Virus Vaccine and a Trivalent Parainfluenza Virus Vaccine in a Pediatric Population	536	9.2
FULGINITI VA, 1969, AM J EPIDEMIOL [33]	Respiratory Virus Immunization: A Field Trial of Two Inactivated Respiratory Syncytial Virus Vaccines	526	9.1
WILSON E, 2023, NEW ENGL J MED [34]	Efficacy and Safety of an mRNA-Based RSV PreF Vaccine in Older Adults	442	110.5
MARCANDALLI J, 2019, CELL [35]	Induction of Potent Neutralizing Antibody Responses by a Designed Protein Nanoparticle Vaccine for RSV	393	49.1
COLLINS PL, 1995, P NATL ACAD SCI USA [36]	Respiratory Syncytial Virus and Metapneumovirus	384	12

## Data Availability

The bibliographic data analyzed in this study were retrieved from the Web of Science Core Collection and Scopus. Access to the original records is subject to the licensing terms of these databases. Processed data supporting the findings are available from the corresponding author upon reasonable request, subject to applicable licensing re-strictions.

## Abbreviations

The following abbreviations are used in this manuscript:

RSV	Respiratory syncytial virus
HRSV	Human respiratory syncytial virus
FI-RSV	Formalin-inactivated respiratory syncytial virus vaccine
WoSCC	Web of Science Core Collection
RPYS	Reference Publication Year Spectroscopy
NIH	National Institutes of Health
NIAID	National Institute of Allergy and Infectious Diseases
GSK	GlaxoSmithKline
mRNA	Messenger ribonucleic acid
mAb	Monoclonal antibody
PRIBA	Preferred Reporting Items for Bibliometric Analysis
MeSH	Medical Subject Headings
